# Insights from comparison of the clinical presentation and outcomes of patients hospitalized with COVID-19 in an Italian internal medicine ward during first and third wave

**DOI:** 10.3389/fmed.2023.1112728

**Published:** 2023-02-01

**Authors:** Andrea Ticinesi, Alberto Parise, Antonio Nouvenne, Nicoletta Cerundolo, Beatrice Prati, Angela Guerra, Domenico Tuttolomondo, Nicola Gaibazzi, Tiziana Meschi

**Affiliations:** ^1^Department of Medicine and Surgery, University of Parma, Parma, Italy; ^2^Geriatric-Rehabilitation Department, Azienda Ospedaliero-Universitaria di Parma, Parma, Italy; ^3^Cardiology Unit, Azienda Ospedaliero-Universitaria di Parma, Parma, Italy

**Keywords:** SARS-CoV-2, B.1.1.7 lineage, respiratory failure, care improvement, geriatric patients, multimorbidity, vaccine

## Abstract

**Background:**

The reasons of variability of clinical presentation of coronavirus disease-19 (COVID-19) across different pandemic waves are not fully understood, and may include individual risk profile, SARS-CoV-2 lineage and seasonal variations of viral spread. The objective of this retrospective study was to compare the characteristics and outcomes of patients admitted with confirmed coronavirus disease-19 (COVID-19) in the same season during the first (March 2020) and the third pandemic wave (March 2021, dominance of SARS-CoV-2 B.1.1.7 lineage) in an internal medicine ward of a large teaching hospital in Italy.

**Materials and methods:**

Data of 769 unvaccinated patients (399 from the first and 370 from the third wave) were collected from clinical records, including symptom type and duration, extension of lung abnormalities on chest computed tomography (CT) and PaO_2_/FiO_2_ ratio on admission arterial blood gas analysis.

**Results:**

Third wave patients were in average younger (median 65, interquartile range [IQR] 55–75, vs. 72, IQR 61–81 years old, *p* < 0.001), with less comorbidities and better pulmonary (CT visual score median 25, IQR 15–40, vs. 30, IQR 15–50, age- and sex-adjusted *p* = 0.017) and respiratory involvement (PaO_2_/FiO_2_ median 288, IQR 237–338, vs. 233, IQR 121–326 mmHg, age- and sex-adjusted *p* < 0.001) than first wave patients. Hospital mortality was lower (19% vs. 36%, *p* < 0.001), but not for subjects over 75 years old (46 vs. 49%). Age, number of chronic illnesses, PCT levels, CT visual score [Odds Ratio (OR) 1.022, 95% confidence interval (CI) 1.009–1.036, *p* < 0.001] and PaO_2_/FiO_2_ (OR 0.991, 95% CI 0.988–0.994, *p* < 0.001), but not the pandemic wave, were associated with mortality on stepwise multivariate logistic regression analysis.

**Conclusion:**

Despite the higher virulence of B.1.1.7 lineage, we detected milder clinical presentation and improved mortality in patients hospitalized during the third COVID-19 wave, with involvement of younger subjects. The reasons of this discrepancy are unclear, but could involve the population effect of vaccination campaigns, that were being conducted primarily in older frail subjects during the third wave.

## 1. Introduction

From February 2020 to May 2021, Italy was strike by three major waves of the coronavirus disease-19 (COVID-19) pandemic, causing peaks of hospital admissions and putting the National Healthcare system under extreme pressure (1). A similar epidemic trend was also observed in other Western countries, especially of the European region, although the magnitude of waves and the response of healthcare systems showed significant differences (1).

Patients who required hospital admission during the first wave were overall characterized by severe respiratory failure, high prevalence of abnormalities on chest imaging and high hospital mortality (2–6). Some reports, however, highlighted differences in the clinical presentation of patients hospitalized for COVID-19 between the earliest and the late phases of the first wave (6, 7). These differences were probably due to improvements in the pre-hospital management and seasonal variations of SARS-CoV-2 transmission and virulence (8). The reduced mortality rates observed in the late phases of the first wave could also depend on improved treatments, particularly the use of intravenous steroids, non-invasive mechanical ventilation and high-flow nasal oxygen delivery devices (9, 10).

Small, but detectable, differences in clinical presentation of COVID-19 cases requiring hospital admission were observed during the second wave in autumn 2020, in comparison with cases from the first wave (11–16). Reduced mortality was also observed, as a result of improved treatment protocols, but not in all studies (17). However, from January 2021 onwards, a novel pandemic wave, sustained by the B.1.1.7 SARS-CoV-2 lineage (alpha variant) rapidly arise. This variant was largely dominant in Italy in March 2021 (18). In other countries, this variant was reported to be associated with increased disease severity and mortality (19, 20). To date, few studies have been focused on the clinical characteristics and outcomes of patients infected during the third pandemic wave in Italy.

Therefore, the aim of this retrospective single-center study was to compare the clinical presentation and outcomes of patients hospitalized with COVID-19 during the same period (March 1–31) of the year 2020 (first wave) and 2021 (third wave) in an internal medicine ward of a teaching hospital in Italy, identifying factors associated with mortality.

## 2. Materials and methods

### 2.1. Patient characteristics and data collection

This study was conducted in an Internal Medicine unit of a large teaching hospital in Northern Italy (Parma University-Hospital), that has been appointed as the main hub for the care of COVID-19 patients of the whole Parma province (approximately 450,000 inhabitants) since the earliest phases of the first wave (21). Two groups of patients hospitalized with COVID-19 in March 2020 and March 2021 were retrospectively enrolled after check for inclusion and exclusion criteria and availability of data on clinical records. The periods of observation were chosen because they corresponded to the first and third wave peaks of the COVID-19 pandemic, respectively, and to avoid confounding by seasonal variations of SARS-CoV-2 virulence and transmission in comparisons.

Only patients aged ≥ 18 years old with SARS-CoV-2 infection confirmed by reverse transcriptase polymerase-chain reaction (RT-PCR) on nasopharyngeal swab performed upon urgent admission were included in the study. Additional inclusion criteria were chest computed tomography (CT) and lab tests including serum C-reactive protein (CRP) performed on the day of admission. Conversely, subjects with missing data on these variables and subjects who were transferred to other wards (i.e., with missing data on outcome) were excluded from the study. The 2021 patients who contracted SARS-CoV-2 infection after having received one or more doses of anti-SARS-CoV-2 vaccine were also excluded.

The records of each participant were reviewed in order to collect demographic data (age and sex), number and types of comorbidities (including hypertension, diabetes, obesity, dyslipidemia, heart diseases, cancer, chronic kidney disease), number of drugs, clinical presentation of COVID-19 (i.e., symptoms and their duration, chest CT abnormalities, vital signs), and the results of lab tests performed on admission, including arterial blood gas analysis, blood cell count, serum creatinine and predicted glomerular filtration rate, D-dimer, CRP and procalcitonin (PCT). The extension of pulmonary infiltrates and abnormalities on chest CT was estimated through calculation of the chest CT visual score, detailed elsewhere (22). Arterial blood oxygen partial pressure and the administered oxygen flow were used to calculate the fractional inspired oxygen saturation (P/F). Data on treatments administered during hospital stay and outcome (survival vs. death) were also collected for all participants.

Ethics Committee approval was obtained (Comitato Etico dell’Area Vasta Emilia Nord, Emilia-Romagna region) under the ID 399/2021/OSS/AOUPR as part of a larger project on clinical and radiological factors associated with mortality in hospitalized COVID-19 patients. All participants, who were contactable by phone or for follow-up reasons, provided written informed consent for participations. For all other cases, the Ethics Committee waived written informed consent collection due to retrospective design of the study.

### 2.2. Statistical analyses

Variables were expressed as median and interquartile range (IQR) or percentages, as appropriate. The characteristics of participants were compared between the 2020 and 2021 groups with the Mann–Whitney or chi-square tests, with adjustment for age and sex with Quade non-parametric ANCOVA (continuous variables) or binary logistic regression (dichotomous variables). The factors independently associated with mortality in both groups were investigated with stepwise multivariate logistic regression models considering participants altogether and after partition by pandemic wave. Age, sex, period of admission, symptom duration, type of symptoms, number of chronic illnesses, obesity, diabetes, hypertension, chronic kidney disease, chronic heart disease, cancer, systolic and diastolic blood pressure, chest CT visual score, P/F on admission arterial blood gas analysis, hemoglobin levels, neutrophil and lymphocyte count, serum creatinine, CRP and PCT were considered as entries in these multivariate models. PCT was either considered as a continuous variable or as classes (class 1: < 0.05 ng/ml; class 2: ≥ 0.05 and < 0.5 ng/ml; class 3: ≥ 0.5 and ≤ 2 ng/ml; class 4: > 2 ng/ml). This partition was applied because, in a study conducted on patients from the first pandemic wave, we demonstrated that admission PCT classes were predictive of survival in oldest old COVID-19 patients (23).

Additional analyses were also made after categorization of participants of both waves by age (< 75 years old vs. ≥ 75 years old), for the known association between age, age-related conditions such as frailty and multimorbidity, and COVID-19 related mortality (6). Finally, the factors independently associated with P/F on admission blood gas analysis were investigated with stepwise multivariate linear regression, for the known prognostic importance of P/F ratio in COVID-19 pneumonia (24).

Analyses were performed with the SPSS statistical package (v. 28, IMB, Armonk, US), considering *p* values < 0.05 as statistically significant.

## 3. Results

We included in this study 399 patients from the first wave and 370 patients from the third wave. Their clinical characteristics are compared in Table 1. Patients from the third wave were younger, and with less comorbidities than those admitted in the first wave. The clinical presentation of COVID-19 was also different, with increased prevalence of diarrhea (17% vs. 6%) and fatigue (34% vs. 11%) as main symptoms, reduced extension of pulmonary involvement on chest CT (visual score median 25, IQR 15–40, vs. 30, IQR 15–50, age- and sex-adjusted *p* = 0.017), improved P/F ratio on blood gas analysis (median 288, IQR 237–338, vs. 233, IQR 121–326 mmHg, age- and sex-adjusted *p* < 0.001). These differences were also mirrored by lower levels of CRP and PCT (Table 1).

**Table 1:** Comparison of the main characteristics of COVID-19 presentation and outcomes between patients admitted during the first wave (March 2020, *n* = 399) and the third wave (March 2021, *n* = 370).

	First wave March 2020 (*n* = 399)	Third wave March 2021 (*n* = 370)	*p*	p^*^
Demography and personal history				
Age, years	72 (61–81)	65 (55–75)	**<0.001**	**–**
Females, %	40	40	0.861	–
Chronic illnesses, number	2 (1–4)	2 (1–3)	**<0.001**	**0.023**
Hypertension, %	61	52	**0.011**	0.630
Diabetes, %	22	18	0.139	0.479
Obesity, %	13	15	0.302	0.833
Dyslipidemia, %	21	19	0.624	0.813
Chronic heart disease, %	24	10	**<0.001**	**<0.001**
Chronic kidney disease, %	6	2	**0.004**	**0.010**
Cancer, %	12	6	**0.006**	**0.021**
Drugs, number	3 (1–6)	2 (0–4)	**<0.001**	**<0.001**
Clinical presentation upon admission				
Duration of symptoms, days	7 (4–10)	6 (3–9)	0.192	**0.017**
Fever, %	89	81	**0.001**	**<0.001**
Cough, %	53	50	0.421	0.095
Dyspnea,%	49	52	0.291	0.165
Fatigue, %	11	34	**<0.001**	**<0.001**
Diarrhea, %	6	17	**<0.001**	**<0.001**
Systolic blood pressure, mmHg	130 (120–140)	130 (120–140)	0.948	0.467
Diastolic blood pressure, mmHg	80 (70–80)	80 (70–80)	0.204	0.151
Chest CT visual score, %	30 (15–50)	25 (15–40)	**0.009**	**0.017**
P/F ratio, mmHg	233 (121–326)	288 (237–338)	**<0.001**	**<0.001**
P/F ratio ≤ 100 mmHg, %	20	5	**<0.001**	**<0.001**
Blood tests on admission				
Hemoglobin, g/dl	13.8 (12.5–14.9)	13.9 (12.8–15.0)	0.499	0.984
Platelet count, 1,000/mm^3^	195 (152–243)	189 (147–244)	0.716	0.278
Neutrophil count, n/mm^3^	4,721 (3338–7,284)	4,871 (3281–6,824)	0.966	0.680
Lymphocyte count, n/mm^3^	893 (630–1,205)	852 (588–1,130)	0.163	**0.032**
Creatinine, mg/dl	0.9 (0.7–1.2)	0.9 (0.7–1.1)	**0.018**	0.377
eGFR, ml/min	80 (57–105)	89 (68–113)	**<0.001**	0.275
D-dimer, ng/ml	922 (600–1,376)	709 (436–1,258)	**<0.001**	**0.022**
CRP, mg/L	106 (50–168)	52 (26–96)	**<0.001**	**<0.001**
PCT, ng/ml	0.17 (0.09–0.50)	0.09 (0.05–0.23)	**<0.001**	**<0.001**
PCT class 1 (<0.05 ng/ml), %	9	22	**<0.001**	**<0.001**
PCT class 4 (>2 ng/ml), %	11	3	**<0.001**	**<0.001**
Treatments and outcomes				
NIV, %	14	28	**<0.001**	**<0.001**
ICU, %	5	13	**<0.001**	**0.002**
Intravenous steroids, %	16	96	**<0.001**	**<0.001**
Hospital death, %	36	19	**<0.001**	**<0.001**
Hospital stay, days	7 (3–12)	14 (9–21)	**<0.001**	**<0.001**

Data expressed as median and interquartile range or percentage. p values calculated with Mann–Whitney or Chi-square test. ^*^p adjusted for age and sex with Quade non-parametric ANCOVA or binary logistic regression. *p* values < 0.05 are indicated in bold.

CT = computed tomography; P/F=PaO_2_/FiO_2_; eGFR = estimated glomerular filtration rate; CRP=C-reactive protein; PCT = procalcitonin; NIV=non-invasive ventilation; ICU=intensive care unit.

In spite of this, patients admitted during the third wave experienced significantly higher rates of non-invasive ventilation (NIV) support (28% vs. 14%) and intensive-care unit (ICU) transferal (13% vs. 5%). However, mortality was significantly lower (19% vs. 36%, age- and sex-adjusted *p* < 0.001).

On a stepwise multivariate logistic regression model (Table 2), age, the number of chronic illnesses, symptom duration, P/F ratio, chest CT visual score and PCT classes were independently associated with hospital mortality. The period of admission (first or third wave) was included in the multivariate model, but was not independently associated with mortality (Table 2).

**Table 2:** Factors associated with hospital mortality on stepwise multivariate logistic regression analysis, considering patients from the first and the third wave altogether.

	Odds ratio	95% Confidence interval	*p*
Age, years	1.061	1.036–1.087	**<0.001**
Chronic illnesses, number	1.348	1.168–1.555	**<0.001**
Duration of symptoms, days	0.939	0.889–0.992	**0.025**
P/F ratio, mmHg	0.991	0.988–0.994	**<0.001**
Chest CT visual score, %	1.022	1.009–1.036	**<0.001**
PCT classes, for each incremental class	1.842	1.300–2.610	**<0.001**

Other variables considered in the model: sex (F vs M), period of admission (third wave vs first wave), fever, cough, dyspnea, fatigue, diarrhea, obesity, diabetes, hypertension, chronic kidney disease, chronic heart disease, cancer, systolic and diastolic blood pressure, hemoglobin, neutrophil count, lymphocyte count, creatinine, C-reactive protein. *p* values < 0.05 are indicated in bold.

P/F=PaO_2_/FiO_2_; CT = computed tomography; PCT = procalcitonin.

In the first wave, age and P/F ratio on admission were the only independent predictors of mortality (Table 3). In the third wave, instead, other factors were involved in addition to age and P/F ratio (Table 3).

**Table 3:** Factors associated with hospital mortality on stepwise multivariate logistic regression analysis, after stratification of participants by COVID-19 wave.

	Odds ratio	95% Confidence interval	WALD	*p*
First wave, March 2020				
Age, years	1.052	1.022–1.083	12.004	**<0.001**
P/F ratio, mmHg	0.988	0.984–0.991	53.351	**<0.001**
Third wave, March 2021				
Age, years	1.094	1.050–1.141	18.176	**<0.001**
Chronic illnesses, number	1.601	1.275–2.010	16.455	**<0.001**
Duration of symptoms, days	0.916	0.843–0.996	4.235	**0.040**
P/F ratio, mmHg	0.991	0.986–0.997	10.509	**0.001**
Chest CT visual score, %	1.036	1.014–1.059	10.088	**0.001**
PCT classes, for each incremental class	2.664	1.431–4.961	9.542	**0.002**

Other variables considered in the model: sex (F vs M), period of admission (third wave vs first wave), fever, cough, dyspnea, fatigue, diarrhea, obesity, diabetes, hypertension, chronic kidney disease, chronic heart disease, cancer, systolic and diastolic blood pressure, hemoglobin, neutrophil count, lymphocyte count, creatinine, C-reactive protein. *p* values <0.05 are indicated in bold.

P/F=PaO_2_/FiO_2_; CT = computed tomography; PCT = procalcitonin.

Supplementary Tables 1 and 2 show a comparison between patients of the two study periods aged < 75 and ≥ 75 years old, respectively. While most differences between the 2020 and 2021 groups, shown in Table 1, were confirmed after stratification by age, mortality showed significant improvement in the 2021 group only in patients < 75 years old (10% vs. 27%, age- and sex-adjusted *p* < 0.001), but not in patients ≥ 75 years old (46% vs. 49%, age- and sex-adjusted *p* = 0.666).

**Figure 1:**
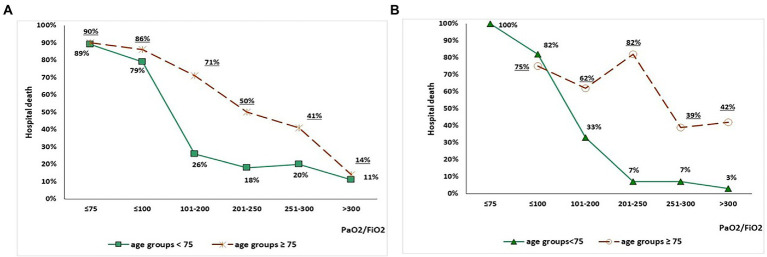
Association between P/F values on admission arterial blood gas analysis and mortality in the 2020 group (panel A) and 2021 group (panel B), stratified by age (< 75 vs. ≥ 75 years old).

The association between P/*F* values on admission arterial blood gas analysis and mortality, according to study period and age range, is depicted in Figure 1. In the 2020 group, increasing P/*F* values were associated with reduced mortality, although mortality remained higher in subjects ≥ 75 years old than in subjects < 75 years old for each P/F class (Figure 1). Conversely, in the 2021 group, mortality in subjects ≥ 75 years old seemed unrelated with P/F values, while a steep decline was observed in patients < 75 years old with P/*F* > 200 mmHg (Figure 1). Table 4 shows the factors independently associated with P/F values in each age class on stepwise multivariate linear regression models. Admission during the third wave was positively associated with P/F in both subjects aged < 75 (standardized *β* = 0.105, *p* = 0.014) and subjects aged 75 or older (standardized *β* = 0.217, *p* < 0.001).

**Table 4:** Stepwise multivariate linear regression models exploring factors independently associated with P/F values in each age class in the studied population of patients from the first and third pandemic wave.

	Standardized beta	*T*	*p*
Model 1, patients < 75 years old			
Age, years	−0.127	−2.788	**0.006**
Chronic illnesses, number	−0.097	−2.091	**0.037**
Presence of dyspnea	−0.120	−2.817	**0.005**
Period of admission (2021 vs. 2020)	0.105	2.479	**0.014**
Chest CT visual score, %	−0.463	−10.441	**<0.001**
Neutrophil count, n/mm^3^	−0.098	−2.287	**0.023**
Model 2, patients ≥ 75 years old			
Presence of dyspnea	−0.234	−3.742	**<0.001**
Period of admission (2021 vs. 2020)	0.217	3.532	**<0.001**
Chest CT visual score, %	−0.353	−5.212	**<0.001**
Neutrophil count, n/mm^3^	−0.136	−2.039	**0.043**

Other variables considered in the model: sex (F vs M), duration of symptoms, fever, cough, fatigue, diarrhea, obesity, diabetes, hypertension, chronic kidney disease, chronic heart disease, cancer, systolic and diastolic blood pressure, hemoglobin, lymphocyte count, creatinine, C-reactive protein, procalcitonin classes. *p* values < 0.05 are indicated in bold. P/F=PaO_2_/FiO_2_.

## 4. Discussion

In this retrospective study, we showed that patients admitted for COVID-19 during the third wave in March 2021 had less severe clinical presentation of the disease and reduced mortality, in comparison with patients admitted during the first wave. Patients from the third wave, however, were younger and had less chronic comorbidities.

These findings are apparently in contrast with experimental and epidemiological data suggesting an increased virulence of the B.1.1.7 SARS-CoV-2 lineage (19, 20, 25), that was responsible for the third COVID-19 wave in Italy (18). COVID-19 severity, however, is significantly influenced by age and multimorbidity (3, 6, 26), and an overwhelming majority of older patients dead with COVID-19 had multimorbidity in their personal history (27). Thus, the involvement of a younger and less comorbid population in the COVID-19 pandemic during the third wave could have masked the increased virulence of the B.1.1.7 lineage.

We can hypothesize that this circumstance may have been the result of the anti-SARS-CoV-2 vaccination campaign, that in Italy was started at the end of December 2020 and was initially focused on healthcare professionals and older subjects with frailty (28). By March 2021, when the third wave arise, a significant rate of the older population had been administered anti-SARS-CoV-2 vaccines, though with significant barriers including social disadvantage (29). These vaccines exhibit the maximum effectiveness against SARS-CoV-2 transmission and protection against severe illness for an interval of 6 months after completion of the primary cycle (30), so that we can assume that a significant portion of the frail older population was protected against COVID-19 by March 2021. A recent study conducted in patients with chronic kidney disease undergoing hemodialysis highlighted that vaccination against SARS-CoV-2 was able to modify COVID-19 severity and reduce hospitalization need even in the presence of a condition of extreme vulnerability (31). The different epidemiological characteristics of patients admitted during the third wave could therefore reflect this phenomenon.

Improvements in hospital management of patients could be also responsible for better outcomes in the third wave. In the 2021 group, 96% of patients had received intravenous steroids during hospital stay, in comparison with just 16% in the first wave (Table 1). Intravenous dexamethasone treatment has rapidly gained the role of cornerstone treatment of COVID-19 related interstitial pneumonia, for its capacity of reducing mortality, oxygen supplementation and ventilatory support need (32). Intravenous remdesivir was also commonly used during the third wave, but not in the first one (33). Interestingly, the higher frequencies of NIV support and ICU treatment detected in the third wave (Table 1) could reflect improved management protocols and better understanding of indications and timing of ventilatory escalation in patients with severe respiratory failure. Better supportive care and evidence-based treatment protocols were recognized as the main factors influencing improved outcomes during the third wave also in another study from Italy (34).

Patients admitted during the third wave, however, had not only better outcomes, but also different clinical pictures on hospital admission. During the third wave, the organization of pre-hospital care was improved in comparison with the abrupt emergence of the pandemic. At a community level, medical teams dedicated to home care of COVID-19 patients were formed, prompting early diagnosis and rationalizing pathways of hospital referral for more severe cases (35, 36). Home treatment protocols could include administration of anti-inflammatory agents, antivirals or, in selected cases, even corticosteroids (37). These aspects could have influenced the clinical presentation of COVID-19 on admission, with less severe pulmonary involvement and better respiratory exchanges. Similar findings were also observed in studies comparing the second (autumn 2020) with the first wave (11–16).

The heterogeneity of clinical presentation of SARS-CoV-2 infection, especially with the emergence of novel lineages, should be also considered (38). This characteristic is particularly emphasized in older patients, where the classical association of fever, cough and dyspnea is found less frequently than in younger subjects (39) and extra-pulmonary involvement is more common (40). The demographical differences between the two groups considered in our study could thus contribute to explain also differences in clinical presentation, and not just in outcomes.

Another remarkable finding of our study concerns the outcomes of patients over 75 years old, that were similar between the two considered waves despite significant differences in clinical presentation and improvements in treatment regimens. Namely, in the 2021 group prognosis of subjects over 75 years old was less dependent on respiratory parameters on admission (Figure 1). We can speculate that this phenomenon may be the effect of an increased burden of frailty, influencing weaker response to treatments during the acute phase of the disease (41). Frailty syndrome is in fact one of the main factors influencing adverse outcomes in older subjects with COVID-19 (42).

Unfortunately, frailty was not systematically assessed in all the participants to our study, preventing to include this variable in the analyses. Further limitations include the retrospective design, the exclusion of a large number of patients hospitalized during the first wave for lack of relevant data, and the absence of SARS-CoV-2 genotypization for identification of lineages on nasopharyngeal swabs.

In spite of this, our study provides important insight on the clinical and epidemiological differences of patients hospitalized during the first and third pandemic waves in Italy, eliminating the possible confounding factor of seasonality in SARS-CoV-2 transmission. Although the differences in clinical presentation and outcomes between the third and the first wave allow to advance several epidemiological hypotheses on the evolution of the COVID-19 pandemic, the circumstance that this is a single-center hospital-based study should be also remarked as a limitation. No data were in fact available on the management of patients in the community setting before hospital arrival and on the clinical characteristics of subjects with COVID-19 who did not require hospitalization.

## 5. Conclusion

Patients hospitalized for COVID-19 during the third pandemic wave were younger and had less comorbidities than patients hospitalized during the first wave. Their clinical presentation was also different, with improved P/F ratio on admission and different symptom distribution. Mortality was also improved, but not in patients older than 75 years old. The reasons of these differences, apparently in contrast with the increased reported severity of the SARS-CoV-2 B.1.1.7 lineage, are unclear. They could be related to the effect of vaccination campaigns in older frail subjects, granting protection against severe disease and favoring the spread of the infection among younger unvaccinated subjects, and improvements in pre-hospital and hospital care.

## Data availability statement

The raw data supporting the conclusions of this article will be made available by the authors upon request, without undue reservation.

## Ethics statement

The studies involving human participants were reviewed and approved by Comitato Etico dell’Area Vasta Emilia Nord, Emilia Romagna Region, Italy. The ethics committee waived the requirement of written informed consent for participation.

## Author contributions

AT, DT, and TM: conception and design. AT, AP, AN, NC, BP, and DT: data collection and interpretation. AG: data analysis. AT: manuscript drafting. NG and TM: critical revision for important intellectual content. All authors contributed to the article and approved the submitted version.

## Conflict of interest

The authors declare that the research was conducted in the absence of any commercial or financial relationships that could be construed as a potential conflict of interest.

## Publisher’s note

All claims expressed in this article are solely those of the authors and do not necessarily represent those of their affiliated organizations, or those of the publisher, the editors and the reviewers. Any product that may be evaluated in this article, or claim that may be made by its manufacturer, is not guaranteed or endorsed by the publisher.
