# Supplementary material for: Insights from comparison of the clinical presentation and outcomes of patients hospitalized with COVID-19 in an Italian internal medicine ward during first and third wave
Source: Front Med (Lausanne). 2023 Feb 1;10:1112728. doi: 10.3389/fmed.2023.1112728 (PMC9928966; doi:10.3389/fmed.2023.1112728)
Supplement: Supplementary file 1 [file Table_1.DOCX]

Insights from comparison of the clinical presentation and outcomes of patients hospitalized with COVID-19 infection in Italy during first and third wave: what caused improved mortality?

Andrea Ticinesi*, Alberto Parise, Antonio Nouvenne, Nicoletta Cerundolo, Beatrice Prati, Angela Guerra, Domenico Tuttolomondo, Nicola Gaibazzi, Tiziana Meschi

*** Correspondence:** Andrea Ticinesi: [andrea.ticinesi@unipr.it](mailto:andrea.ticinesi@unipr.it)

# Supplementary Tables

- 1. **Supplementary Table 1**

Comparison of the main characteristics of COVID-19 presentation and outcomes between patients aged <75 years old admitted during the first wave (March 2020, n=228) and the third wave (March 2021, n=276).

|  | **First wave**  **March 2020**  **(n=228)** | **Third wave March 2021**  **(n=276)** | **p** | **p*** |
| --- | --- | --- | --- | --- |
| **Demography and personal history** | | | |  |
| Age, years | 63 (54-70) | 60 (52-68) | **0.004** | **-** |
| Females, % | 36 | 37 | 0.832 | - |
| Chronic illnesses, number | 2 (1-3) | 1 (0-3) | **<0.001** | **0.002** |
| Hypertension, % | 52 | 44 | 0.077 | 0.352 |
| Diabetes, % | 21 | 15 | 0.069 | 0.187 |
| Obesity, % | 19 | 16 | 0.452 | 0.360 |
| Dyslipidemia, % | 20 | 17 | 0.372 | 0.708 |
| **Clinical presentation upon admission** | | | |  |
| Duration of symptoms, days | 7 (5-10) | 7 (4-9) | 0.088 | 0.059 |
| Fever, % | 93 | 86 | **0.024** | **0.011** |
| Cough, % | 58 | 52 | 0.167 | 0.098 |
| Dyspnea,% | 47 | 51 | 0.453 | 0.411 |
| Fatigue, % | 12 | 37 | **<0.001** | **<0.001** |
| Diarrhea, % | 7 | 16 | **0.003** | **0.003** |
| Chest CT visual score, % | 30 (15-45) | 25 (15-40) | 0.155 | 0.224 |
| P/F ratio, mmHg | 255 (153-344) | 295 (243-340) | **<0.001** | **0.004** |
| P/F ratio ≤100 mmHg, % | 18 | 5 | **<0.001** | **<0.001** |
| **Blood tests on admission** | | | |  |
| Haemoglobin, g/dl | 13.9 (12.4-14.9) | 14.0 (13.1-15.0) | **0.022** | **0.015** |
| Platelet count, 1000/mm^3^ | 202 (165-250) | 189 (152-243) | 0.139 | 0.073 |
| Neutrophil count, n/mm^3^ | 4595 (3273-7171) | 4792 (3219-6673) | 0.963 | 0.912 |
| Lymphocyte count, n/mm^3^ | 948 (674-1204) | 874 (631-1169) | 0.160 | 0.087 |
| Creatinine, mg/dl | 0.9 (0.7-1.1) | 0.8 (0.7-1.0) | **0.035** | 0.110 |
| D-dimer, ng/ml | 765 (514-1217) | 627 (409-987) | **0.001** | **0.006** |
| CRP, mg/L | 103 (46-164) | 47 (23-91) | **<0.001** | **<0.001** |
| PCT, ng/ml | 0.15 (0.07-0.33) | 0.07 (0.04-0.17) | **<0.001** | **<0.001** |
| PCT class 1 (<0.05 ng/ml), % | 13 | 25 | **0.002** | **0.002** |
| PCT class 4 (>2 ng/ml), % | 9 | 2 | **0.001** | **0.006** |
| **Treatments and outcomes** | | | |  |
| NIV, % | 23 | 27 | 0.313 | 0.212 |
| ICU, % | 9 | 15 | 0.055 | **0.040** |
| Intravenous steroids, % | 15 | 97 | **<0.001** | **<0.001** |
| Hospital death, % | 27 | 10 | **<0.001** | **<0.001** |
| Hospital stay, days | 8 (4-13) | 13 (9-19) | **<0.001** | **<0.001** |

Data expressed as median and interquartile range or percentage. P values calculated with Mann-Whitney or chi-square test. *P adjusted for age and sex with Quade non-parametric Ancova or binary logistic regression. P values <0.05 are indicated in bold.

CT=Computed Tomography; P/F=PaO_2_/FiO_2_; CRP=C-Reactive Protein; PCT=Procalcitonin; IL-6=Interleukin-6; NIV=Non-Invasive Ventilation; ICU=Intensive Care Unit.

- 1. **Supplementary Table 2**

Comparison of the main characteristics of COVID-19 presentation and outcomes between patients aged ≥75 years old admitted during the first wave (March 2020, n=171) and the third wave (March 2021, n=94).

|  | **First wave**  **March 2020**  **(n=171)** | **Third wave March 2021**  **(n=94)** | **p** | **p*** |
| --- | --- | --- | --- | --- |
| **Demography and personal history** | | | |  |
| Age, years | 82 (79-87) | 82 (78-86) | 0.733 | **-** |
| Females, % | 46 | 47 | 0.852 | - |
| Chronic illnesses, number | 3 (2-5) | 4 (2-4) | 0.870 | 0.843 |
| Hypertension, % | 74 | 77 | 0.656 | 0.628 |
| Diabetes, % | 24 | 27 | 0.580 | 0.598 |
| Obesity, % | 4 | 12 | **0.019** | **0.028** |
| Dyslipidemia, % | 22 | 27 | 0.377 | 0.388 |
| **Clinical presentation upon admission** | | | |  |
| Duration of symptoms, days | 5 (3-8) | 5 (1-9) | 0.055 | 0.051 |
| Fever, % | 85 | 66 | **<0.001** | **<0.001** |
| Cough, % | 45 | 43 | 0.698 | 0.657 |
| Dyspnea,% | 50 | 57 | 0.264 | 0.240 |
| Fatigue, % | 8 | 23 | **<0.001** | **<0.001** |
| Diarrhea, % | 5 | 19 | **<0.001** | **<0.001** |
| Chest CT visual score, % | 30 (20-50) | 25 (15-40) | **0.027** | **0.021** |
| P/F ratio, mmHg | 208 (110-310) | 279 (220-327) | **<0.001** | **<0.001** |
| P/F ratio ≤100 mmHg, % | 23 | 5 | **<0.001** | **0.001** |
| **Blood tests on admission** | | | |  |
| Haemoglobin, g/dl | 13.7 (12.5-15.0) | 12.9 (10.8-14.4) | **<0.001** | **0.001** |
| Platelet count, 1000/mm^3^ | 183 (139-234) | 187 (132-254) | 0.833 | 0.839 |
| Neutrophil count, n/mm^3^ | 4815 (3451-7525) | 5343 (3430-7522) | 0.533 | 0.513 |
| Lymphocyte count, n/mm^3^ | 810 (596-1206) | 759 (480-1081) | 0.117 | 0.120 |
| Creatinine, mg/dl | 1.0 (0.8-1.4) | 1.0 (0.8-1.3) | 0.753 | 0.666 |
| D-dimer, ng/ml | 1121 (710-2201) | 1187 (651-2209) | 0.901 | 0.918 |
| CRP, mg/L | 107 (56-178) | 72 (44-113) | **<0.001** | **<0.001** |
| PCT, ng/ml | 0.23 (0.10-0.69) | 0.16 (0.08-0.46) | **0.045** | **0.047** |
| PCT class 1 (<0.05 ng/ml), % | 4 | 12 | **0.012** | **0.018** |
| PCT class 4 (>2 ng/ml), % | 14 | 4 | **0.017** | **0.021** |
| **Treatments and outcomes** | | | |  |
| NIV, % | 2 | 32 | **<0.001** | **<0.001** |
| ICU, % | 0 | 6 | **<0.001** | **-** |
| Intravenous steroids, % | 17 | 94 | **<0.001** | **<0.001** |
| Hospital death, % | 49 | 46 | 0.665 | 0.666 |
| Hospital stay, days | 6 (3-11) | 15 (11-25) | **<0.001** | **<0.001** |

Data expressed as median and interquartile range or percentage. P values calculated with Mann-Whitney or chi-square test. *P adjusted for age and sex with Quade non-parametric Ancova or binary logistic regression. P values <0.05 are indicated in bold.

CT=Computed Tomography; P/F=PaO_2_/FiO_2_; CRP=C-Reactive Protein; PCT=Procalcitonin; IL-6=Interleukin-6; NIV=Non-Invasive Ventilation; ICU=Intensive Care Unit.
